# Long-Range Distributed Solar Irradiance Sensing Using Optical Fibers

**DOI:** 10.3390/s20030908

**Published:** 2020-02-08

**Authors:** Regina Magalhães, Luis Costa, Sonia Martin-Lopez, Miguel Gonzalez-Herraez, Alejandro F. Braña, Hugo F. Martins

**Affiliations:** 1 Dpto. de Electrónica, Universidad de Alcalá, 28805 Alcalá de Henares (Madrid), Spain; luis.duarte@uah.es (L.C.); Sonia.martinlo@uah.es (S.M.-L.); miguel.gonzalezh@uah.es (M.G.-H.); 2 Grupo de Electrónica y Semiconductores, Dpto. de Física Aplicada, Universidad Autónoma de Madrid, 28049 Madrid, Spain; alejandro.brana@uam.es; 3 Instituto de Óptica, Consejo Superior de Investigaciones Científicas, 28006 Madrid, Spain; h.fidalgo@uah.es

**Keywords:** distributed sensing, optical fiber sensors, optical radiation, optical time domain reflectometry, photothermal effects, rayleigh scattering, solar irradiance, solar energy

## Abstract

Until recently, the amount of solar irradiance reaching the Earth surface was considered to be a steady value over the years. However, there is increasing observational evidence showing that this quantity undergoes substantial variations over time, which need to be addressed in different scenarios ranging from climate change to solar energy applications. With the growing interest in developing solar energy technology with enhanced efficiency and optimized management, the monitoring of solar irradiance at the ground level is now considered to be a fundamental input in the pursuit of that goal. Here, we propose the first fiber-based distributed sensor able of monitoring ground solar irradiance in real time, with meter scale spatial resolutions over distances of several tens of kilometers (up to 100 km). The technique is based on an optical fiber reflectometry technique (CP-ϕOTDR), which enables real time and long-range high-sensitivity bolometric measurements of solar radiance with a single optical fiber cable and a single interrogator unit. The method is explained and analyzed theoretically. A validation of the method is proposed using a solar simulator irradiating standard optical fibers, where we demonstrate the ability to detect and quantify solar irradiance with less than a 0.1 W/m^2^ resolution.

## 1. Introduction

The Sun is the main source of energy of our planet. Therefore, even a slight change in its output energy can make a huge impact in terrestrial climatic conditions, affecting many fields going from environmental sciences [1], to climatology [2] and energy [3]. In these areas, surface solar irradiance is often considered to be an essential input variable, with special and particular interest in the energy domain. Owing to the increasing number of solar-based systems implemented worldwide, the monitoring and forecasting of surface solar irradiance is considered to be decisive for the development of successful solar energy technologies, and fundamental for an optimized use of solar energy in the future [4]. One of the current limitations standing against the growing exploration of solar energy-based technologies is caused by the intrinsic nature of solar energy itself, fluctuating stochastically in time due to the existence of irregular meteorological patterns. By achieving high-quality monitoring and consequent forecasting of solar irradiance behavior, it may be possible to develop smart-grids (by compensating variations of the input solar energy), and also help to reduce the cost of integrating solar plants into the existing power grids, achieving a better grid management [5]. 

In the last years, a huge effort has been made in increasing the accuracy of ground solar irradiance estimation methods and in achieving optimized solar radiation forecasts, namely through the use of satellite images [6,7,8]. However, ground solar irradiance measurement is still the most accurate method for characterizing the solar irradiance at a given site [8]. Despite the increasing availability of databases of ground solar irradiance achieved through different measurement networks, the sparsity of the measurements provided is still very limiting [4,8,9,10,11]. Historic sequences of measured ground solar radiation are still scarcely available for a given spot where a solar-based system is planned, making it challenging to accurately predict performances. This is a problematic scenario, since the accurate knowledge of solar radiation at the Earth’s surface is essential for developing energy efficient buildings, solar components, and sophisticated solar energy management. Fundamentally, the development of these applications directly requires information on the temporal evolution of the meteorological conditions influencing the system [4].

Concerning the monitoring of ground solar irradiance in real time over large perimeters, clear limitations can be found in the existing technology. In addition to the scarce number of ground measured points provided by most technologies, when considering the currently available remote sensing techniques based on geostationary satellites, the temporal resolutions provided can only range from a few minutes to several hours [12], and the typical spatial resolutions available are of the order of a few km [1,4]. Therefore, the development of distributed solar radiation sensing technology capable of measuring across large distances (i.e., tens of kilometers) with meter-scale spatial resolution and second-scale readout could be very beneficial. In this paper, we demonstrate that optical fiber technology may provide this very beneficial tool to the solar energy field, providing highly-sensitive meter-resolved maps of ground solar irradiance across perimeters of tens of km, in real time, and with kHz sampling rates. These maps could help to increase unprecedentedly the density of the solar irradiance databases and networks available, possibly benefiting as well some of the satellite-based statistical models which also require ground solar radiation data and information. The need for this type of technology becomes even more evident when considering the extensively reported errors occurring in the interpolation and extrapolation of solar radiation, involved when using particular station measurements as representatives of nearby sites [13,14,15,16,17,18]. 

Ultimately, distributed solar irradiance sensors could help several applications in monitoring solar irradiance over large distances where it represents a limiting factor, such as Dynamic Line Rating (DLR) forecasting [19,20], and electricity grid management [21], where the rapidly increasing penetration of solar power in the electric grid is directly affecting the efficiency of these systems. They could also be installed in photovoltaic plants to monitor variations of solar irradiance across the field with high sensitivity (helping to achieve an efficient and optimized use of solar energy), or in public illumination management where a single detector is typically used to activate a large number of street lights.

Nevertheless, there are some challenges in developing distributed solar irradiance sensing technology. In distributed sensing, an optical fiber cable serves as a continuous data transmission and sensing element, in which light-matter interactions occurring along the fiber allow the retrieval of information about the local properties at any given position in the cable (e.g., temperature [22,23,24], pressure [25,26,27], or strain [28,29,30]). Depending on the distributed sensing method, the exploited light-matter interactions can be originated by Rayleigh, Brillouin, or Raman scattering, which are mostly temperature or strain dependent [31]. Distributed sensing techniques only require a single optical fiber cable connected to a single interrogation unit, allowing this way to replace thousands of independent detectors which would be needed to monitor the same perimeter. Distributed Fiber Optic Sensors (DFOS) enable therefore an increase in space and weight efficiency, as well as a reduction in the overall complexity, energy consumption, and cost-per-sensor, particularly when a large number of detectors is intended. Due to these features and considering their ability to perform continuous large-scale position-resolved measurements of different physical quantities, DFOS have been receiving a remarkable amount of attention in the last years in a wide diversity of areas, such as civil engineering, energy, and seismology [32,33,34,35,36]. The scattering effects present in DFOS are mostly temperature and strain dependent, therefore the measurement of a different parameter (like irradiance in this case) typically requires a transducing layer to convert the target physical quantity into a strain or temperature change in the fiber core. In the case of solar irradiance, a possible solution might come by developing a photothermal-based distributed sensor, whose temperature changes in the presence of an external irradiance. However, for this to be possible, a sufficiently high temperature sensitivity is needed in order to accurately detect tiny variations in the surface solar radiation. Considering that typical temperature resolutions in conventional DFOS (e.g., Brillouin-based DFOS) are around 1 K [37], this idea becomes impractical, since the amount of radiation needed to reach a measurable change in the cable temperature would be too high.

Recently, an advanced optical fiber reflectometry technique using chirped-pulses in phase-sensitive optical time domain reflectometry (CP-ϕOTDR) has been demonstrated to achieve sub-mK resolutions in distributed temperature detection, achieving fast (kHz) and reliable measurements over distances of up to 100 km with meter-scale spatial resolutions [38,39,40]. With these advances, it is now possible to develop a feasible and practical distributed surface solar irradiance sensor using optical fibers. The central idea of the system is based on integrating two optical fiber cables: one of them with a high absorptive coating to translate an optical radiation absorption into a temperature change in the fiber core, and the other one with a low absorption coefficient operating as a non-absorptive reference. By monitoring the temperature difference between the two cables with the high temperature resolution provided by CP-ϕOTDR, it is possible to obtain a feasible distributed measurement of solar radiation along tens of km in real time. 

Here we show the proof-of-concept of a distributed ground solar irradiance sensor based on CP-ϕOTDR. The demonstration is made by monitoring the temperature behavior of a black and a white optical fiber cables using an intensity-tunable solar simulator under AM1.5G light spectrum. This demonstration is an important milestone in solar radiation technology, opening the door for important advances in many industries, directly or indirectly related to solar energy.

## 2. Theoretical Principles

### 2.1. Principle of Bolometry

The principle of bolometry relies on measuring the power of incident electromagnetic radiation by monitoring the temperature of a material with a known absorption coefficient. Accordingly, the development of a bolometer requires the implementation of an absorptive material, at temperature *T*, thermally connected to a heat sink, at temperature *T*_0_. Since the temperature of the absorptive material depends on the incident irradiance, in the case of no incident irradiance, $E_{inc}=0$, both materials have the same temperature, $T=T_{0}$. However, in the presence of an external irradiance in the system, i.e., at the condition $E_{inc}>0$, the absorptive element absorbs an optical power, $P_{abs}=E_{inc}\cdot A_{inc}\cdot \alpha$, which is proportional to the incident irradiance, the absorber’s area of radiation incidence, *A_inc_*, and its absorption coefficient, *α*. Therefore, *T* increases to a higher value than *T*_0_, becoming $T>T_{0}$. Assuming a small temperature difference between the elements, $T-T_{0}$, the power dissipation between the absorber and the thermal sink can be described following Newton’s law of cooling [41]:

(1) $$P_{dissipated}=h\cdot A\cdot (T-T_{0}),$$

where *h* refers to the heat transfer coefficient between the elements, and *A* to the heat transfer surface area. In these conditions, the rate equation describing the balance of energy occurring in the bolometer is given by:

(2) $$dE_{gained}-dE_{lost}=c\cdot m\;dT,$$

where the constants *c* and *m* refer to the absorber’s specific heat and the absorber’s mass, respectively. 

On one hand, the energy gained in the system is directly dependent on the optical power absorbed, increasing for higher values of external irradiance. On the other hand, the energy lost depends on the dissipated optical power from the absorber to the heat sink, which also increases for higher absorber temperatures. Accordingly, the terms $dE_{gained}$ and $dE_{lost}$ in Equation (2) are given by:

(3) $$dE_{gained}=P_{abs}\;dt=E_{inc}\cdot A_{inc}\cdot \alpha \;dt,$$

(4) $$dE_{lost}=P_{dissipated}\;dt=h\cdot A\cdot (T-T_{0})\;dt.$$

By replacing these relations in the rate equation described in Equation (2), we arrive to:

(5) $$E_{inc}\cdot A_{inc}\cdot \alpha \;dt-h\cdot A\cdot (T-T_{0})\;dt=c\cdot m\;dT,$$

which can be rewritten in a simpler form, defining $a=-\frac{E_{inc}\cdot A_{inc}\cdot \alpha +h\cdot A\cdot T_{0}}{c\cdot m}$ and $b=-\frac{h\cdot A}{c\cdot m}$:

(6) $$\frac{dT}{dt}=bT-a.$$

The solution to this differential equation can be easily derived, and it has the form:

(7) $$T(t)=\frac{E_{inc}\cdot A_{inc}\cdot \alpha +h\cdot A\cdot T_{0}}{\;h\cdot A}+K\cdot \exp\left(\frac{-h\cdot A}{c\cdot m}t\right).$$

By considering the initial condition of the system (at t=0) when both elements have the same temperature, $T(t=0)=T_{0}$, we can arrive to the value of *K*, which is given by:

(8) $$K=\frac{-E_{inc}\cdot A_{inc}\cdot \alpha }{h\cdot A}.$$

Finally, by replacing Equation (8) in Equation. (7), we arrive to the general rate equation describing the temporal evolution of the bolometer, i.e., the evolution of the absorber’s temperature with relation to the external irradiance:

(9) $$T(t)=T_{0}+\left(\frac{E_{inc}\cdot A_{inc}\cdot \alpha }{h\cdot A}\right)\cdot \left(1-\exp\left(\frac{-h\cdot A}{c\cdot m}t\right)\right).$$

Furthermore, as the absorber’s temperature increases due to absorption, so does the dissipation, which is proportional to the temperature difference between the elements (as seen from Equations (3) and (4)). Consequently, as the system evolves, it eventually reaches a thermal equilibrium state, where $P_{abs}=P_{dissipated}$. By equaling the respective expressions, $E_{inc}\cdot A_{inc}\cdot \alpha =h\cdot A\cdot (T-T_{0})$, or simply by considering a large value of *t* in Equation (9), the following relation can be derived at the thermal equilibrium state:

(10) $$E_{inc}≃\frac{\left(T-T_{0})\cdot (h\cdot A\right)}{\left(A_{inc}\cdot \alpha \right)}=\frac{\left(T-T_{0}\right)}{¢},$$

where the constant *¢* is given by the parameters of the system. This way, given a bolometric system where the necessary parameters are known and the thermal equilibrium condition is verified, an incident irradiance *E_inc_* can be calculated using the relation obtained in Equation (10).

### 2.2. Developing a Long-Range Distributed Fiber-Based Bolometer

The principle of bolometry described above can be implemented using distributed optical fiber-based technology. The core idea of the methodology here proposed is aimed at solving the most important practical limitation of a long-range distributed bolometer: the temperature of the heat-sink *T*_0_ (ambient temperature along the fiber installation) is not known a priori. With the solution of this problem in mind, the proposed fiber-based bolometer works by monitoring the temperature difference of two optical fibers with different coatings (different absorption coefficients). In this case, the measurement of an incident irradiance, *E_inc_*, can be achieved for an arbitrary and unknown ambient temperature, *T*_0_. 

The mathematical formalism behind this idea is described below. Two optical fibers with different optical absorption coefficients, *α*_1_, *α*_2_, (and/or different heat transfer coefficients *h*_1_, *h*_2_), are placed in the same environment and under the same irradiance. In this case, the thermal equilibrium state for fiber 1 and fiber 2 is reached at different temperatures, *T*_1_, *T*_2_:

(11) $$E_{inc}=\frac{\left(T_{1}-T_{0})\cdot (h_{1}\cdot A\right)}{A_{inc}\cdot \alpha_{1}}=\frac{\left(T_{1}-T_{0}\right)}{{¢}_{1}}\Leftrightarrow T_{0}=T_{1}-(E_{inc}\cdot {¢}_{1}),$$

(12) $$E_{inc}=\frac{\left(T_{2}-T_{0})\cdot (h_{2}\cdot A\right)}{A_{inc}\cdot \alpha_{2}}=\frac{\left(T_{2}-T_{0}\right)}{{¢}_{2}}\Leftrightarrow T_{0}=T_{2}-(E_{inc}\cdot {¢}_{2}).$$

By replacing Equation (11) in Equation (12), it is derived that:

(13) $$E_{inc}=\frac{\left(T_{2}-T_{1}\right)}{{¢}_{2}-{¢}_{1}}.$$

**Figure 1:**
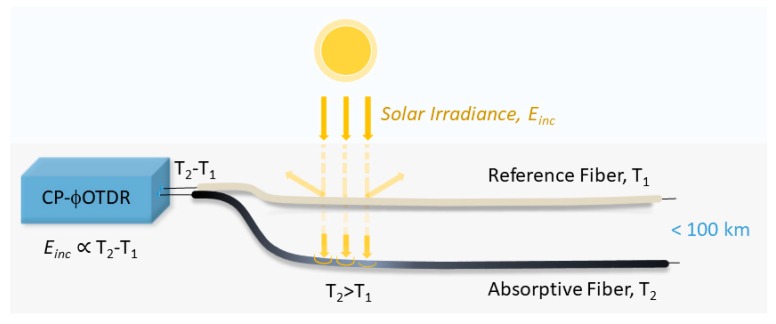
Representation of the operation of the proposed long-range distributed solar irradiance sensor.

As shown in Equation (13), in a fiber-based bolometer, an incident irradiance *E_inc_* can be measured for an arbitrary unknown external temperature value, *T*_0_, by simply measuring the difference between the temperature shifts obtained for two fibers with different absorption coefficients. Additionally, *¢*_1_ and *¢*_2_ can accommodate for different values of *α* and *h*, and are not needed individually, but only their difference ${¢}_{2}-{¢}_{1}$. This difference can be easily measured for any given set of fibers, by applying a known irradiance to the bolometric system, and by measuring the resulting $T_{2}-T_{1}$ in equilibrium. Therefore, the proposed sensor only requires a single initial calibration. A representation of the operation of the proposed distributed fiber-based bolometer can be seen in Figure 1. 

Considering modern industrial processes for the fabrication of optical fibers, the features of the coatings such as the material and the thickness are not expected to vary significantly along the sensing range of the fiber. We can therefore assume that the absorption coefficient, the incidence area, and heat transfer area are identical for different positions of the fiber and along its range. As such, ${¢}_{2}-{¢}_{1}$ is also expected to present negligible variability, provided even installation conditions along the sensing range. A different issue concerns variation of the area of incidence (*A_inc_*) due to environmental issues external to the fiber. In fact, punctual obstructions may realistically be faced at different positions of the fibers in a realistic installation, including dust, snow, shadows, etc. In these cases, the incidence area, *A_inc_*, for instance, would change locally and affect the value of ${¢}_{2}-{¢}_{1}$, and the solar irradiance would be prevented for reaching the fiber. However, such issue would affect as well any radiation sensor at the ground level (punctual or distributed), as the problem relies on the fact that radiation is not reaching the sensor. Therefore, all these scenarios must be properly considered and minimized at the installation phase, as it is also done with any bolometer system which relies on irradiance absorption. In general, the installation should be designed in order to allow the maximum exposition of the fiber to the effects of sun radiation, while also ensuring minimal variation in the heat transfer characteristics of the optical cable (along the distance and across the time).

### 2.3. Distributed Fiber Temperature Interrogator: CP-ϕOTDR

The performance (sensitivity and dynamic response) of the bolometer will be directly linked to those of the used fiber interrogator, which therefore has a critical role in the system. In order to accurately measure the incident irradiance along the fiber-based bolometer system, we propose a distributed sensing technique based on ϕOTDR with linearly chirped pulses (CP-ϕOTDR) [38].

In conventional ϕOTDR, highly coherent optical pulses are injected into an optical fiber, and by monitoring the resulting Rayleigh backscattered light it is possible to measure a perturbation along the fiber. The ϕOTDR signal depends on multiple and random contributions given by scattering centers along the fiber, and also on the intensity and phase profile of the pulses. Given a certain temperature (ΔT) or strain (Δε) perturbation in the fiber, the effective refractive index, *n*, will be modified by an amount Δn, changing the ϕOTDR trace. This change, Δn, can be compensated by shifting the pulse frequency, Δν, which in turn changes the ϕOTDR pattern. By finding the Δν which allows to recover the original trace (reference trace), the amount Δn suffered by the fiber can be measured. Assuming a small refractive index change (Δn≪n), the necessary Δν to compensate for a given Δn can be derived from [38]:

(14) $$\frac{\Delta n}{n}=\frac{\Delta \nu }{\nu_{o}}\approx -0.78\cdot \Delta \varepsilon \approx -(6.92\cdot 10^{-6})\cdot \Delta T,$$

where $\nu_{o}$ is the central frequency of the pulse. Based on this principle, ϕOTDR systems have been shown to allow for the detection of temperature/strain [37], and birefringence [42] with high resolutions in many different applications.

**Figure 2:**
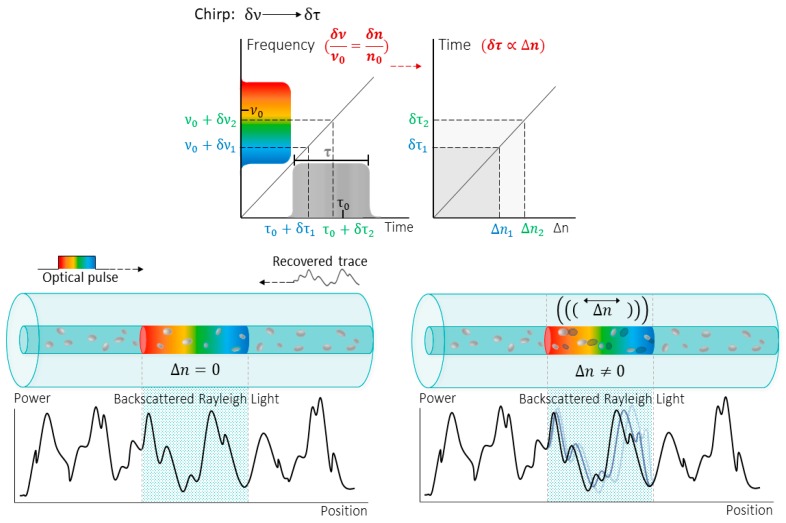
Working principle of chirped-pulses in phase-sensitive optical time domain reflectometry (CP-ϕOTDR): A linear frequency chirped pulse is sent into the optical fiber. Scattering centers along the fiber elastically scatter the light, being part of it guided in the opposite direction of the pulse probe with random amplitude and phase. The resulting optical power from the interference of all backscattered light creates the Backscattered Rayleigh trace. This recovered trace can be considered the fingerprint of the fiber, and it changes as the optical path distance (OPD) is changed locally within the fiber (changes in the refractive index or length due to an external stimulus). As stated in Equation (14), a frequency detuning may be used to compensate the change in the OPD, retrieving the original optical power trace (before the perturbation). With the linear chirp, the frequency detuning is mapped into a time delay within the pulse window, so the optical power is recovered as a time delay proportional to the refractive index change.

In CP-ϕOTDR, the pulse-to-pulse frequency scanning is replaced by a single linearly chirped pulse probe signal [38,39]. Given a chirped-pulse bandwidth much larger than the transform-limited pulse bandwidth, any external temperature or strain induced changes in the fiber optical path will translate into a local time shift (at the corresponding optical trace position). Therefore, by finding the time-delay shift which allows to recover the original trace (directly related to a certain Δν), the amount Δn suffered by the optical fiber can be measured. As a result, the measurement is converted into a time-delay estimation problem (see Figure 2).

This method provides a linear relationship with the applied stimulus, contrary to the conventional direct detection ϕOTDR technique which fails at providing a true monotonic and linear relation to the perturbation. Consequently, CP-ϕOTDR sensors are not only able of detecting and localizing an external stimulus, but they are also able of quantifying them with high accuracy. In addition, CP-ϕOTDR ensures a consistent value of sensitivity along the length of the fiber, without being affected by the fading points (which are positions with nearly no visibility affecting conventional ϕOTDR traces) [38,39]. Therefore, CP-ϕOTDR sensors are extremely appealing for applications that need to quantify a certain physical parameter over large distances and that require a similar measuring quality in all points along the fiber, being widely implemented in areas going from security to seismology [33,34]. Currently, CP-ϕOTDR has shown to reach sub-mK resolutions over ranges of up to 100 km with metric spatial resolution and sampling rates of the order of 1 kHz [39], reaching more recently a record dynamic strain sensitivity of $10^{-12}\varepsilon /\sqrt{Hz}$ (equivalent to $10^{-6}K/\sqrt{Hz}$) [43].

## 3. Materials and Methods

### 3.1. CP-ϕOTDR:Distributed Temperature Sensing Scheme

**Figure 3:**
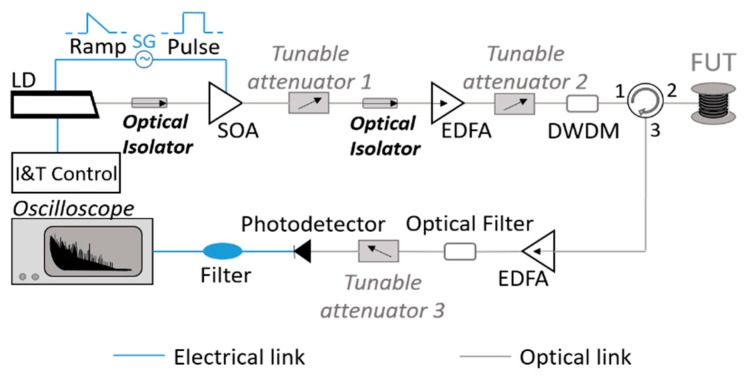
Chirped-pulse phase-sensitive OTDR setup (CP-ϕOTDR): acronyms are explained within the text.

The experimental setup used in the proposed proof-of-concept demonstration of a distributed solar irradiance sensor is depicted in Figure 3. The sensor is a CP-ϕOTDR interrogator [38,39], whose operation has been already described in Section 2. 

The CP-ϕOTDR interrogation scheme consists of a first section which is responsible for generating linearly-chirped pulses, and a second part responsible for the detection of the Rayleigh backscattered light. The first step in the generation of linearly-chirped pulses is done using a Laser Diode (LD) working in continuous wave emission, driven by a temperature and current controller (I&T). The laser is then modulated in current, using an electrical ramp signal given by a Signal Generator (SG). The formation of linearly-chirped pulses is completed by synchronizing the laser current modulation gated with a square-wave to the driver of a Semiconductor Optical Amplifier (SOA). Following this process, the pulses are amplified using an Erbium-doped Fiber Amplifier (EDFA), and their power is controlled using a tunable attenuator to avoid non-linear effects in the optical fiber. A Dense Wavelength-division Multiplier (DWDM) is also implemented in the setup to reduce the amplified spontaneous emission (ASE) produced by the EDFA, filtering the optical signal. After this section, the pulses are sent into the Fiber Under Test (FUTs) using an optical circulator. The backscattered light generated during the process is then collected, amplified and respectively filtered before reaching the photodetector. Finally, it is recorded using a high-speed digitizer. 

**Figure 4:**
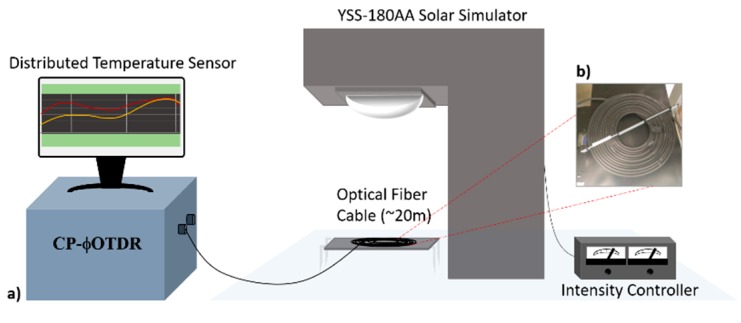
(**a**) Experimental setup proposed for the application and detection of solar irradiance; (**b**) Standard black telecommunications fiber cable, implemented as the fiber under test.

In this particular experiment, the CP-ϕOTDR measurements were performed with 60 ns pulses, corresponding to a 6-meter spatial resolution. These pulses were linearly chirped with ~0.4 GHz of total pulse spectral content. In this configuration, the detection scheme enabled a temperature sampling period of 0.5 ms (2 kHz), achieving an overall temperature resolution of ~1 mK. With this setup, several standard single-mode fibers (SMF) were tested. The first two fibers presented an identical structure (250 μm diameter with an acrylate polymer coating), with the only difference being the color of the outer layer (coating)—one white and one black. A standard telecommunications fiber cable (~1.2 cm diameter with a black thermoplastic jacket) was also tested aiming at verifying the feasibility of the sensor in real implementation conditions (see Figure 4b).

### 3.2. Solar Irradiance Application Scheme

In order to validate our proposed distributed sensor, we used a YSS-180AA solar simulator from NPC Inc. able of replicating the spectrum of the sun (see Figure 4). The simulator consists of a high-pressure xenon lamp used as a light source, employed in this case to irradiate several sets of optical fibers with a maximum irradiation intensity of 1 kW/m^2^, and with a standard AM 1.5 G spectral distribution. This solar simulator in commonly used as a cell tester in solar cell production line, providing a thermally insulated platform to place the samples (in this case, the optical fibers), and an intensity controller to control the irradiance provided by the light source. Several optical fibers were placed under the tunable solar simulator under different values of irradiance, while their temperature behavior was monitored in real time with the distributed temperature sensing technique.

## 4. Results

### 4.1. Proof-of-Concept

**Figure 5:**
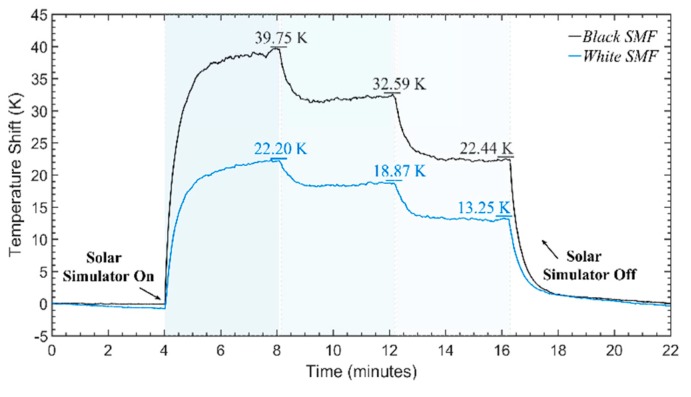
Temperature behavior obtained with the white fiber (**blue trace**), and with the black fiber (**black trace**), under ~4-min solar irradiance cycles of 0 W/m^2^, 1000 W/m^2^, 750 W/m^2^ 500 W/m^2^, and 0 W/m^2^, at room temperature.

**Figure 6:**
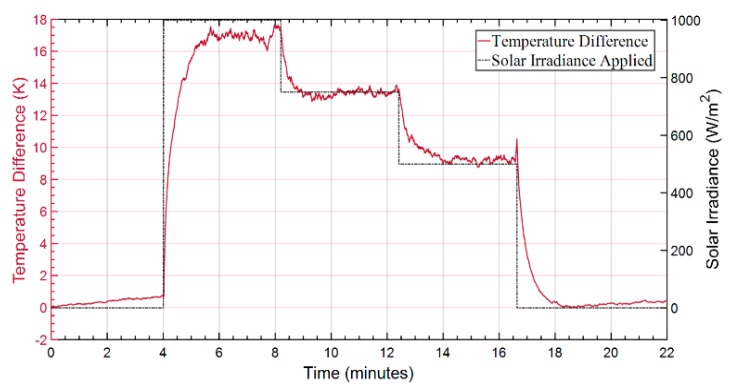
Temperature difference obtained between the black fiber and white fiber traces from Figure 5 vs. the solar irradiance applied.

In the proof-of-concept demonstration, the black and white coated SMFs were characterized under varying cycles of solar irradiance with a duration of a few minutes (~4 min) in each stage. For this purpose, the fibers were placed on top of the thermally insulating platform (under the solar lamp), wrapped in concentric circles reaching ~20 m in length (> than the 2-meter spatial resolution). The sequence of irradiance cycles was applied to the fibers using the solar simulator was the following: (1) Einc = 0 W/m^2^, (2) Einc = 1000 W/m^2^, (3) Einc = 750 W/m^2^, (4) Einc =500 W/m^2^, and (5) Einc = 0 W/m^2^. Since these fibers had a cross section area of ≈ 0.005 m^2^ over 20 m of fiber length, the radiation power reaching the fibers varied between 0 W up to 5 W. The temperature behavior obtained by the CP-ϕOTDR for each of the fibers can be observed in Figure 5, and the temperature difference in Figure 6. 

In Figure 5, the black fiber was observed to reach the thermal equilibrium state at higher temperatures than the white fiber, an expected behavior following Equations (11) and (12), and considering the higher absorption coefficient of the black fiber. The temperature difference between the two fibers (Figure 6) was observed to be proportional to the incident solar irradiance values applied. This fully demonstrates the operation of the sensor, according to Equation (13). 

Since in this case the applied irradiance is a known value (within an uncertainty of ~5 W/m^2^), a simple calibration can be done in order to find the value of 1/(¢2-¢1) for these particular fibers. Using the results of two experiments (done under the same conditions), the values of 1/(¢2-¢1) ≈ 56.08 (Wm^−2^K^−1^) and 1/(¢2-¢1) ≈ 50.20 (Wm^−2^K^−1^) could be calculated for these fibers. Taking the mean value obtained from the calibrations, 1/(¢2-¢1) ≈ 53.14 (Wm^−2^K^−1^), a mean error of 4% can be estimated for the results in Figure 5 and Figure 6. While a smaller deviation of 2.8% and 2.3% can be measured for stages (3) and (4), a deviation of 6.8% can be measured for stage (2). Indeed, note that the thermal equilibrium state of the system is not fully reached in the 4 min for all the cycles: thus, a small but noticeable temperature drift is observed, particularly at the starting and ending stages (1) and (5), with *E_inc_* = 0, and during stage (2). Due to the same reason, an uncertainty was also added to the 1/(¢2-¢1) estimation from the calibrations. Nevertheless, although que equilibrium state is not perfectly reached to allow the determination of the exact constant values, the results fully demonstrate the operation and feasibility of the proposed solar irradiance sensor. The results show the ability to detect and measure typical solar irradiance values with a resolution of 0.06 W/m^2^, which can be found from the resolution of the CP-ϕOTDR (1 mK), or from the sensitivity obtained in Figure 6.

**Figure 7:**
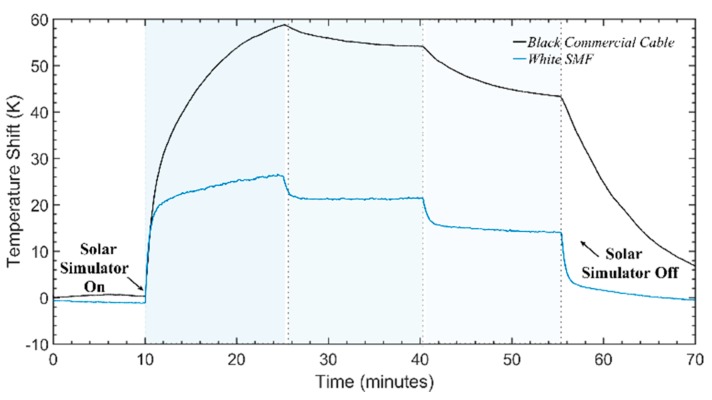
Temperature behavior obtained the white fiber (**blue trace**) and with a commercial black cable (**black trace**), under ~15-min solar irradiance cycles of 0 W/m^2^, 1000 W/m^2^, 750 W/m^2^ 500 W/m^2^, and 0 W/m^2^, at room temperature.

Following this demonstration, the same experiment was done using this time a standard black telecommunications optical fiber cable (see Figure 4b), and the same white SMF as reference. This experiment was done in order to test how a ready-to-implement cable would respond under the same environmental conditions as the previous case, demonstrating not only the operation of the proposed distributed sensor, but its feasibility in field applications as well. The standard black telecommunications optical fiber cable had an outer diameter of ~1.2 cm with two optical fibers inside, presenting a much slower thermal behavior (i.e., a higher thermal capacity) when compared to the standard optical fiber. Therefore, the irradiance cycles in this experiment were longer (~15 min), while the solar radiation values were the same as in the previous case. The temperature shift obtained for both fibers can be seen in Figure 7. 

Looking at the results, although the thickness of the fiber cable is much greater than the thickness of a standard fiber (absorption happening furtherer from the fiber core), and thus the response is much slower, the optical fiber cable is still able to detect the presence of an external solar irradiance with high sensitivity. The temperature difference between the traces is also proportional to the applied irradiance, a behavior expected from Equation (13), thus qualitatively demonstrating the feasibility of using this system as a distributed bolometer for solar radiation. When comparing to the results of Figure 5 however, it was observed that the thermal equilibrium was not reached, and a further quantitative analysis of the system was not possible (we were limited due to time constraints with the experiment). It should be noted that reaching the equilibrium state is not intrinsically required as the theoretical model of the introduction can be used to fit data of temperature variations under non-equilibrium thermal states (i.e., during transient times). However, such discussion is out of the scope of this paper.

Nevertheless, the sensitivity of a standard telecommunications fiber cable was also demonstrated and validated, consisting of a high potential solar irradiance sensor for ready field implementation. Considering the robustness of these cables, they can easily be installed in solar farms or energy facilities to monitor solar irradiance reaching the ground in real time.

### 4.2. 24-h Temperature Experiment

Compared to traditional (e.g., Brillouin, Raman) temperature sensors, CP-ϕOTDR provides a 1000-fold improvement in terms of measurement time (tens of seconds to ms) and sensitivity (~1 K [37] to ~mK [38,39,40]). On the other hand, the sensor provides temperature variation measurements, instead of absolute temperature measurements. In the case of solar irradiance, we have to account for several hours of continuous measurements during daylight exposition times, which on average have the duration of 12 h (ultimately reaching ~22 h in some locations). 

Considering the target applications of solar irradiance monitoring, a 24 h measure and symmetry is expected. By “setting” the temperature difference between the two used fibers to zero during nighttime (when it is known that *E_inc_* = 0), the system can be “recalibrated” once a day, eventually allowing to subtract the effect of any fixed light sources in the vicinity of the fiber. This allows for long term stability of the system, as long as the system is demonstrated to be reliable over a period of 24 h. Therefore, the performance characterization of the CP-ϕOTDR during an operation time of 24 h emerges as a critical parameter to demonstrate the feasibility of the proposed sensor for practical applications. 

**Figure 8:**
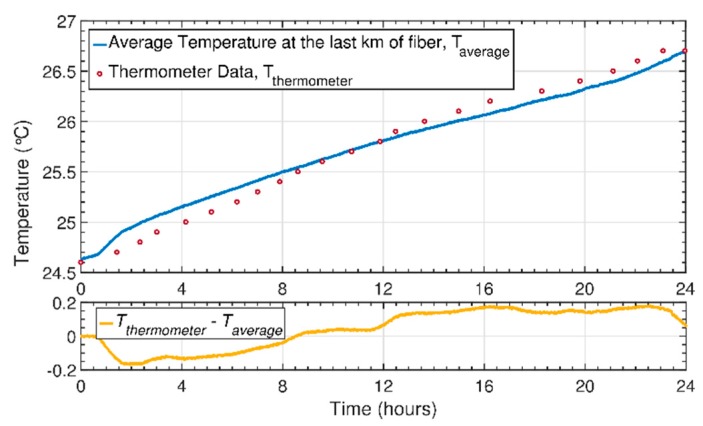
Temporal evolution of the average temperature in the last kilometer of fiber vs. the registered temperature with a thermometer over a period of 24 h. Below, the difference between the two traces is represented, after a linear interpolation was performed to the thermometer data.

With the previous point in mind, we assessed our sensor and the cumulative temperature errors after a large period of time, by performing a 24 h continuous temperature experiment with a CP-ϕOTDR interrogator connected to 4 km of standard optical fiber. The temperature behavior was monitored across all the length of the fiber (a sample each 20 m, in order to reduce the amount of data collected), while the temperature in the vicinity of the fiber was also monitored using a calibrated digital thermometer. The results in Figure 8 show the average temperature behavior at the last kilometer of fiber, paired with the data from the thermometer obtained over the same period of time. As it can be observed, there is a good correlation between the average temperature behavior over the last km of fiber and the data collected with a thermometer during the entire 24 h. Looking at the difference between the traces displayed in Figure 8 (below), a maximum deviation of 200 mK can be found at the end of the fiber (towards the end of the experiment). Although this error is of the order of the thermometer uncertainty (0.1 K), the deviation can be owed to a real temperature difference, since the different positioning of the thermometer probe and the fiber in the room can lead to slight real temperature differences between the sensors. Regardless, since the thermometer temperature uncertainty is two orders of magnitude above that of the CP-ϕOTDR, we further investigated the measured temperature fluctuations by comparing the temperature response in different positions of the fiber. 

**Figure 9:**
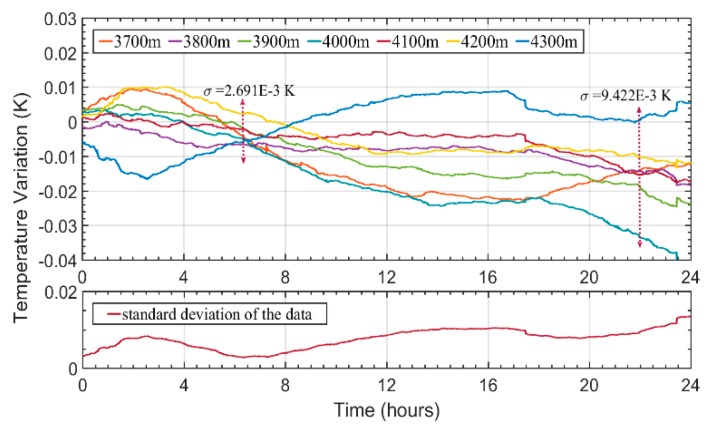
Difference between the temperature traces at the last positions of the fiber and the average temperature behavior. Below, the evolution of the standard deviation between the above traces is depicted.

For this analysis, we took the difference between each temperature trace at the last km of fiber and the average temperature data, studying the standard deviation between the measured points along the 24 h. The results can be seen in Figure 9, as well as the evolution of the standard deviation between the temperature traces. An increasing deviation between the data towards the end of the experiment can be observed, reaching 15 mK after a period of 24 h. Since this value is one order of magnitude lower than the 200 mK deviation seen in Figure 8, this strongly suggests that the deviation is owed to a real temperature difference between the sensors. Therefore, through this characterization we were able to demonstrate that CP-ϕOTDR systems are suitable for performing temperature measurements over large periods of time (as intended in the proposed applications), without substantial cumulative errors interfering with the measurements.

In summary, the proposed system has been characterized in terms of feasibility for long term temperature sensing measurements. The characterization was performed by comparing the CP-ϕOTDR response with the digital thermometer data, showing a good consistency in temperature along the several positions of the fiber. The feasibility of the proposed fiber-based solar irradiance sensor has been therefore fully-addressed and demonstrated for long temperature measurements, and its operation shown in accordance with the theory throughout the results. Based on this demonstration, the proposed distributed sensor has a high potential for integration in applications which could benefit from a real-time monitoring of solar irradiance at a ground level and over large distances, overcoming the clear lack of technology existing in this field.

## 5. Conclusions

In this work, a proof-of-concept demonstration of the first optical fiber-based distributed ground solar irradiance sensor has been proposed and implemented. The method is based on sensitively measuring the temperature difference between two optical fibers with different emissivities, which act as a transducer for the incident radiation, independently of the external temperature. With this method, we have shown that it is possible to obtain high-sensitivity absolute measurements of solar irradiance in a distributed way (less than 0.1 W/m^2^ resolution), with high sampling rates (well below 1 s) and in real time, due to the features of the interrogation scheme provided (CP-ϕOTDR). As a proof-of-concept demonstration, two standard optical fibers with different coatings (one white and one black) were monitored under the irradiance of a tunable solar simulator able of replicating the spectrum of the sun, demonstrating the operation of the proposed sensor with less than a 7% error. A robust standard telecommunications optical fiber cable with a black coating was also interrogated following this method, showing a good sensitivity as well in the presence of solar radiation, and demonstrating, therefore, the feasibility of the technique for installation in harsh field environments. The performance of the CP-ϕOTDR was also characterized over a 24-h temperature experiment (considering the daily exposition sunlight period), readily demonstrating to consist in a suitable technique for the performance of long term measurements without the influence of significant cumulative errors. Owing to the characteristics of the interrogation technique, the proposed distributed bolometer has potential to be readily implemented over distances of up to 100 km, with metric (1-10 m) spatial resolution, and using a single interrogation unit [43]. 

Overall, the demonstrated multi-point detector has a high potential for integration in several solar energy-related fields that could benefit from the distributed monitoring of surface solar irradiance. Among them, applications such as DLR forecasting, public illumination management, or photovoltaic industries could benefit from the first fully distributed solution in ground solar irradiance sensing, since it overcomes the problem of a single data point provided by conventional technologies at a ground level, and the temporal and spatial resolutions provided by the existing remote sensing techniques. Additionally, it has been proven to work with already industrially available standard cables, offering a reliable and ready-to-implement solution. 

Lastly, considering the growing exploration of solar energy systems worldwide, the demonstrated sensor can also consist of an important step towards the development of solar energy smart-grids, since it might be possible to compensate variations of the input solar energy using real time high sensitive ground solar irradiance data. This type of sensor can therefore be an important tool in reducing the cost of integrating solar plants into the existing power grids, helping to achieve a better grid management and a greater efficiency in solar energy systems in the future.
